# Mild behavioral impairment linked to progression to Alzheimer’s disease and cortical thinning in amnestic mild cognitive impairment

**DOI:** 10.3389/fnagi.2022.1051621

**Published:** 2023-01-04

**Authors:** Eun Jin Yoon, Jun-Young Lee, Seyul Kwak, Yu Kyeong Kim

**Affiliations:** ^1^Memory Network Medical Research Center, Seoul National University, Seoul, South Korea; ^2^Department of Nuclear Medicine, SMG-SNU Boramae Medical Center, Seoul, South Korea; ^3^Department of Psychiatry, SMG-SNU Boramae Medical Center, Seoul, South Korea; ^4^Department of Psychiatry, Seoul National University College of Medicine, Seoul, South Korea; ^5^Department of Medical Device Development, Seoul National University College of Medicine, Seoul, South Korea; ^6^Department of Psychology, Pusan National University, Busan, South Korea; ^7^Department of Nuclear Medicine, Seoul National University College of Medicine, Seoul, South Korea

**Keywords:** mild behavioral impairment, mild cognitive impairment, Alzheimer’s disease, neuropsychiatric symptoms, magnetic resonance imaging, cortical thickness

## Abstract

**Background:**

Mild behavioral impairment (MBI) is a neurobehavioral syndrome characterized by later life emergence of sustained neuropsychiatric symptoms, as an at-risk state for dementia. However, the associations between MBI and a risk of progression to Alzheimer’s disease (AD) and its neuroanatomical correlates in mild cognitive impairment (MCI) are still unclear.

**Method:**

A total 1,184 older adults with amnestic MCI was followed for a mean of 3.1 ± 2.0 years. MBI was approximated using a transformation algorithm for the Neuropsychiatric Inventory at baseline. A two-step cluster analysis was used to identify subgroups of individuals with amnestic MCI based on profiles of 5 MBI domain symptoms (decreased motivation, affective dysregulation, impulse dyscontrol, social inappropriateness, abnormal perception/thought content). A Cox regression analysis was applied to investigate differences in the risk of progression to AD between subgroups. A subset of participants (*n* = 202) underwent 3D T1-weighted MRI scans at baseline and cortical thickness was compared between the subgroups of amnestic MCI patients.

**Result:**

The cluster analysis classified the patients into 3 groups: (1) patients without any MBI domain symptoms (47.4%, asymptomatic group); (2) those with only affective dysregulation (29.4%, affective dysregulation group); (3) those with multiple MBI domain symptoms, particularly affective dysregulation, decreased motivation and impulse dyscontrol (23.2%, complex group). Compared to the asymptomatic group, the complex group was associated with a higher risk of progression to AD (hazard ratio = 2.541 [1.904–3.392], *p* < 0.001), but the affective dysregulation group was not (1.214 [0.883–1.670], *p* = 0.232). In cortical thickness analysis, the complex group revealed cortical thinning bilaterally in the inferior parietal, lateral occipital, lateral superior temporal, and frontopolar regions compared with the affective dysregulation group.

**Conclusion:**

The multiple co-occuring MBI domains in individuals with amnestic MCI are associated with a higher risk of progression to AD and cortical thinning in temporal, parietal and frontal areas. These results suggest that evaluation of MBI could be useful for risk stratification for AD and appropriate intervention in MCI individuals.

## Introduction

Mild behavioral impairment (MBI) is a diagnostic construct that describes later life acquired, sustained and impactful neuropsychiatric symptoms (NPS) of any severity that cannot be better accounted for by other formal medical and psychiatric nosology. MBI represents the neurobehavioral axis of pre-dementia risk states, as a complement to the neurocognitive risk axis represented by mild cognitive impairment (MCI). MBI symptoms are divided into the 5 domains: decreased motivation, affective dysregulation, impulse dyscontrol, social inappropriateness, and abnormal perception or thought content, which incorporates a range of NPS (Ismail et al., 2016). Recent studies have consistently identified that MBI is associated with cognitive decline and risk of progression to dementia. MBI was associated with decline in attention and working memory function in cognitively unimpaired participants (Creese et al., 2019), and cognitive and functional decline in older adults with subjective cognitive decline (SCD) (Ismail et al., 2021). Also, older adults with MBI had a higher conversion rate to dementia than those without MBI (Ruthirakuhan et al., 2022) or a comparator group consisting of psychiatric conditions (Taragano et al., 2018; Matsuoka et al., 2019). Neuroimaging studies have identified relationships between MBI and Alzheimer’s disease (AD)-related biomarkers. The total score of the MBI checklist (MBI-C) was correlated with thickness in the entorhinal cortex and the volume of the hippocampus in individuals with SCD or MCI (Matuskova et al., 2021). In cognitively unimpaired participants, higher MBI-C total scores predicted higher Amyloid-ß (Aß) PET uptake in the frontal and subcortical areas (Lussier et al., 2020). In Aß-positive cognitively unimpaired subjects, higher MBI-C total scores were associated with higher tau-PET uptake in the entorhinal cortex and hippocampus as well as CSF p-tau levels (Johansson et al., 2021). This body of evidence highlights the importance of MBI as an early clinical manifestation of dementia, particularly AD.

Although MCI is widely regarded as an intermediate stage between cognitively normal aging and dementia (mainly AD), it is a heterogeneous condition with varied cognitive impairment profiles and clinical outcomes (Petersen, 2004; Manly et al., 2008). Thus, it is essential to identify modifiable risk factors of AD for risk stratification and appropriate intervention. However, in MCI, little work has been done on the relationship of NPS and progression to dementia and structural neuroimaging changes in terms of presence of MBI. A recent study identified a MBI domain of abnormal perception/thought content was associated with a risk of conversion to AD in patients with MCI (Yokoi et al., 2019). This study evaluated the effects of each MBI domain on the risk of dementia, but individual MBI domains can co-occur and be correlated with each other. For example, depression and apathy are included in different MBI domains, but co-occur in many cases (Marin et al., 1994). In another recent study with combined group of individuals with SCD or MCI, MBI-C scores were negatively correlated with the hippocampus and entorhinal cortex atrophy associated with early AD pathology, suggesting that MBI is potentially useful in further detection of individuals at-risk of developing AD. However, this study used just 5 selected regions of interest, thus there would be possibilities of missing out more widespread structural changes related with MBI (Matuskova et al., 2021).

Therefore, in this study, we explored clusters of patients with amnestic MCI (aMCI) based on interrelationship of MBI domains using a two-step cluster analysis, which is a suitable and reliable clustering method for clinical data (Gelbard et al., 2007; Kent et al., 2014). The patients followed longitudinally and then the differences in the risk of AD progression between resulting subgroups were evaluated by means of a survival analysis. To identify the neuroanatomical correlates of MBI that help to understand structural mechanisms of the associations between MBI and risk of AD, we also analyzed the cortical thickness and subcortical volume differences between the subgroups in the whole brain at baseline. We hypothesized that the risk of progression from aMCI to AD would vary by group membership, with a higher risk in groups with complex or severe MBI domain symptoms. Further we hypothesized that MBI would be associated with the brain atrophy in the frontal region or anterior cingulate cortex in terms of NPS (Rosenberg et al., 2015) as well as the parietal and temporal areas included in AD cortical signature (Dickerson et al., 2009).

## Materials and methods

### Participants

A total 1,184 individuals with aMCI who visited SMG-SNU Boramae Medical Center or Dongjak-Gu Center for Dementia between 2012 and 2020 were included in the present study. All participants were administered the Korean mini-mental state examination (MMSE), the clinical dementia rating (CDR), and the Consortium to Establish a Registry for AD for Koreans (CERAD-K) neuropsychological battery (Lee et al., 2002) by the trained clinical psychologists at baseline and subsequent follow-ups. The diagnosis of the AD and aMCI were based on the criteria of the National Institute of Neurological and Communicative Disorders and Stroke and AD and Related Disorders Association (NINCDS-ADRDA) and Petersen’s criteria, respectively (McKhann et al., 1984; Petersen, 2004). For this study, inclusion criteria were: (1) 60 to 85 years of age at baseline; (2) at least one follow-up; (3) more than 1 year of a follow-up time; and (4) administration of neuropsychiatric inventory questionnaire (NPI-Q) at their basal visit. Exclusion criteria were: (1) history of head trauma, brain tumor, stroke, intellectual disability, or any severe medical, neurological, or psychiatric illness affecting cognitive function; (2) a history of alcohol or drug abuse; and (3) progression to dementia other than AD such as dementia with Lewy body, vascular dementia, and frontotemporal lobe dementia. All participants gave written informed consent according to the Declaration of Helsinki, and the protocol was approved by the Institutional Review Board of SMG-SNU Boramae Medical Center (IRB No.30–2020-181).

### Assessment of mild behavioral impairment

MBI was assessed in accordance with criterion of the International Society to Advance Alzheimer’s Research and Treatment - Alzheimer’s Association (ISTAART-AA) research diagnostic criteria for MBI (Ismail et al., 2016) using the NPI-Q (Cummings et al., 1994). Ten NPI items were used to approximate whether individuals met the five MBI domains criteria as follows; (1) decreased motivation (NPI-Q: apathy/indifference); (2) affective dysregulation (NPI-Q: depression/dysphoria, anxiety, elation/euphoria); (3) impulse dyscontrol (NPI-Q: agitation/aggression, irritability/liability, aberrant motor behavior); (4) social inappropriateness (NPI-Q: disinhibition); and (5) abnormal perception or thought content (NPI-Q: delusions, hallucinations). The NPI neurovegetative items of sleep and appetite changes are not reflected in the ISTAART-AA MBI criteria and have been excluded. The NPI-Q was rated by clinical psychologists based on the semi-structured interview administered to the patients’ informants or caregivers. Presence of at least one NPI symptom within a specific MBI domain meant that the domain criteria were met. If at least one of the five MBI domains was present, participants classified as MBI-positive. Although MBI criteria requires a 6-month reference range, 1-month reference range of NPI-Q was used to ascertain MBI in the present study.

### MRI acquisition and preprocessing

Among the 1,184 aMCI patients, 214 patients were scanned using a Philips Achieva 3-T MRI scanner (Philips Medical Systems, Netherlands) at baseline. A high-resolution T1-weighted spoiled gradient recalled 3D MRI sequence was obtained covering the whole brain (224 slices, TR = 9.9 ms, TE = 4.6 ms, FA = 8°, FOV = 220 × 220 mm^2^, voxel size = 0.98 × 0.98 × 1 mm^3^). The FreeSurfer imaging analysis suite (http://surfer.nmr.mgh.harvard.edu/; version 7.1.0) was used to estimate cortical thickness and subcortical volumes. The details of these procedures have been extensively described in prior publications (Fischl and Dale, 2000). Briefly, skull stripping, transformation to Talairach space, segmentation of subcortical white and gray matter structures, intensity normalization, tessellation of the gray/white matter boundaries, automated topology correction, and surface deformation following intensity gradients to optimally place the gray/white (white matter surface) and gray/CSF (pial surface) borders that most accurately define the transition to the other tissue class. Segmented volumes were visually inspected, and the appropriate manual corrections were performed. Twelve participants were excluded from the following statistical analyses because of the low quality of FreeSurfer output. The data was smoothed on the surface with a 10-mm FWHM Gaussian kernel. The volumes of subcortical structures including the bilateral caudate nucleus, putamen, pallidum, nucleus accumbens, hippocampus, amygdala, and thalamus were also calculated.

### Statistical analysis

To test whether meaningful subgroups could be classified based on participants’ neuropsychiatric profiles, we performed a two-step cluster analysis. The dichotomous variables (0 = absence, 1 = presence) of 5 MBI domains were used as inputs. In the first step, participants were pre-clustered into small groups by constructing a clustering feature tree. During the second step, sub-clusters from the first step were entered as inputs and grouped into best number of clusters according to the agglomerative hierarchical clustering method. Cluster distance and cluster criterion were calculated using log-likelihood and Schwarz’s Bayesian criterion (BIC), respectively. The differences in demographical and clinical data between the resulting clusters were analyzed with one-way analysis of variance with the Tukey post-hoc test and χ^2^ test as appropriate. Univariate Cox regression analyses were conducted with age at baseline, gender, years of education, MMSE at baseline, short form of geriatric depression scale (SGDS) at baseline and presence of family history of dementia to identify predictors associated with a risk of progression to AD. A positive family history was defined as one or more first-degree relatives with documented dementia. Significant predictors from univariate analyses were included as covariates in subsequent multivariate Cox regression analyses to evaluate the differences in the risk of progression to AD between resulting subgroups of aMCI patients. The analyses were performed using SPSS 28.0.

Vertex-wise comparisons of the subgroups of aMCI patients were carried out using general linear model. Age, gender, years of education, MMSE and SGDS at baseline were included as covariates of no interest and cluster-wise correction using Monte Carlo simulation was applied (cluster-wise probability = 0.01, α= 0.05). Volumes of subcortical structures were analyzed in the same manner as the vertex-wise analyses using general linear model. The results of volume analyses were considered as statistically significant when surviving the *p* < 0.05, corrected for multiple comparisons using false discovery rate.

## Results

The demographic and clinical characteristics of the participants at baseline are presented in Table 1. Among the 1,184 participants, 623 participants (52.6%) were MBI-positive. The most prevalent MBI domain was affective dysregulation (47.3%). Social inappropriateness and abnormal perception/thought content were the least prevalent domains in these participants (4.4%). During follow-ups, total 284 participants developed AD (24%). Patients with aMCI who progressed to AD had older age, lower MMSE and higher SGDS at baseline and shorter years of education compared with those who did not progress to AD or other dementias. Sex and the presence of family history of dementia were not significantly different between the two groups divided according to whether they progressed to AD. Mean conversion time from aMCI to AD was 2.7 ± 1.8 years. Demographic and clinical variables of the two groups are described in Table 2.

**Table 1:** Demographic and clinical characteristics of the study participants at baseline.

	Mean ± SD (Range)/Frequency (%)
Numbers	1,184
Age, years	73.5 ± 5.7 (60–80)
Female:Male	731:453
Education, years	8.4 ± 4.5 (0–23)
MMSE	22.8 ± 3.4 (9–30)
SGDS	6.1 ± 3.5 (0–15)
Family history of dementia	141 (11.9)
MBI-positive	623 (52.6)
Decreased motivation	150 (12.7)
Affective dysregulation	560 (47.3)
Impulse dyscontrol	168 (14.2)
Social inappropriateness	52 (4.4)
Abnormal perception/thought content	52 (4.4)
Follow-up, years	3.1 ± 2.0 (0.9–9.4)
Progression to AD	284 (24)

SD, standard deviation; MMSE, mini-mental state examination; SGDS, short form of geriatric depression scale; MBI, mild behavioral impairment; AD, Alzheimer’s disease.

**Table 2:** Demographic and clinical characteristics of the study participants classified according to whether they progressed to Alzheimer’s disease.

	Stable aMCI^a^	aMCI-AD^b^	*p*-value
Numbers	900	284	
Age, years	72.8 ± 5.5 (60–85)	75.6 ± 5.6 (60–85)	< 0.001
Female:Male	555: 345	176: 108	0.944
Education, years	8.7 ± 4.4 (0–23)	7.7 ± 4.7 (0–18)	0.002
MMSE	23.1 ± 3.2 (9–30)	21.7 ± 3.7 (10–30)	< 0.001
SGDS	6.0 ± 3.4 (0–15)	6.5 ± 3.8 (0–15)	0.038
Family history of dementia	100 (11.1)	41 (14.4)	0.141
Follow-up^c^, years	3.2 ± 2.0 (0.9–9.4)	2.7 ± 1.8 (0.9–9.3)	< 0.001

Variables are presented mean ± standard deviation (range) or frequency (%). aMCI, amnestic mild cognitive impairment; AD, Alzheimer’s disease; MMSE, mini-mental state examination; SGDS, short form of geriatric depression scale.^a^aMCI participants who did not progress to AD dementia or other dementias.^b^aMCI participants who progressed to AD.^c^Time from baseline to last assessment for the stable aMCI group and conversion time from aMCI to AD for the aMCI-AD group.

### Subgroups of participants according to the cluster analysis of MBI domains

The two-step cluster analysis resulted in a three-cluster solution with a good Silhouette Coefficient equal to 0.8. The three clusters solution gave the highest value for the ratio of distance measure of 2.95 and low BIC value of 1682.08. The first cluster was constituted by patients without any MBI domain symptoms (n = 561, 47.4%) and second cluster was constituted by patients with only affective dysregulation (n = 348, 29.4%). Therefore, these patient groups were referred as “asymptomatic” and “affective dysregulation,” respectively. Third cluster included rest of the aMCI patients (*n* =  275, 23.2%). Most of the participants including in the third cluster revealed symptoms in multiple MBI domains (1 domain, *n* = 45 (16.4%); 2 domains, *n* = 130 (47.3%); 3 domains, *n* = 74 (26.9%); 4 or 5 domains, *n* = 27 (9.4%)). When the multiple domains coexist, 91.8% (*n* = 212) of the participants have affective dysregulation domain with other MBI domains. Prevalent cases were that affective dysregulation coexisted with decreased motivation (*n* = 59, 21.5%), or impulse dyscontrol (*n* = 40, 14.5%), or both (*n* = 43, 15.6%). The third group of patients was referred as “complex.” Demographic and clinical variables of the 3 groups of aMCI patients are described in Table 3. The complex group reveled differences in age and duration of follow-ups compared with the affective dysregulation group, and higher rate of progression to AD compared with asymptomatic and affective dysregulation groups. The affective dysregulation group consisted of more females than the other groups. Two symptomatic groups had higher SGDS scores than the asymptomatic group.

**Table 3:** Demographic and clinical characteristics of the study participants classified based on profiles of mild behavioral impairment domain symptoms.

	Asymptomatic (1)	Affective dysregulation (2)	Complex (3)	*p*-value^a^			
				3 groups	1 vs. 2	1 vs. 3	2 vs. 3
numbers	561	348	275				
Age, years	73.3 ± 5.5 (60–85)	73.0 ± 5.8 (60–85)	74.3 ± 5.8 (60–85)	0.022	0.713	0.071	0.021
Female:Male	313: 248	249: 99	169:106	< 0.001	< 0.001	0.120	0.008
Education, years	8.7 ± 4.5 (0–23)	8.1 ± 4.2 (0–18)	8.2 ± 4.9 (0–20)	0.092	0.134	0.221	0.994
MMSE	22.9 ± 3.2 (10–30)	22.7 ± 3.6 (9–30)	22.6 ± 3.6 (12–30)	0.541	0.622	0.645	1.00
SGDS	4.5 ± 2.6 (0–15)	7.8 ± 3.0 (0–15)	7.3 ± 4.1 (0–15)	< 0.001	< 0.001	< 0.001	0.114
Family history of dementia	60 (10.7)	40 (11.5)	41 (14.9)	0.201	0.708	0.079	0.129
Follow-up, years	3.1 ± 1.9 (0.9–9.2)	3.3 ± 2.1 (0.9–9.4)	2.8 ± 1.9 (0.9–9.3)	0.012	0.467	0.081	0.009
Progression to AD	98 (17.5)	77 (22.1)	109 (39.6)	< 0.001	0.083	< 0.001	< 0.001

Variables are presented mean ± standard deviation (range) or frequency (%). ^a^one-way analysis of variance with the Tukey *post-hoc* test for continuous variables and *χ*^2^ test for nominal variables. MMSE, mini-mental state examination; SGDS, short form of geriatric depression scale; AD, Alzheimer’s disease.

### Risk of progression to AD

**Figure 1:**
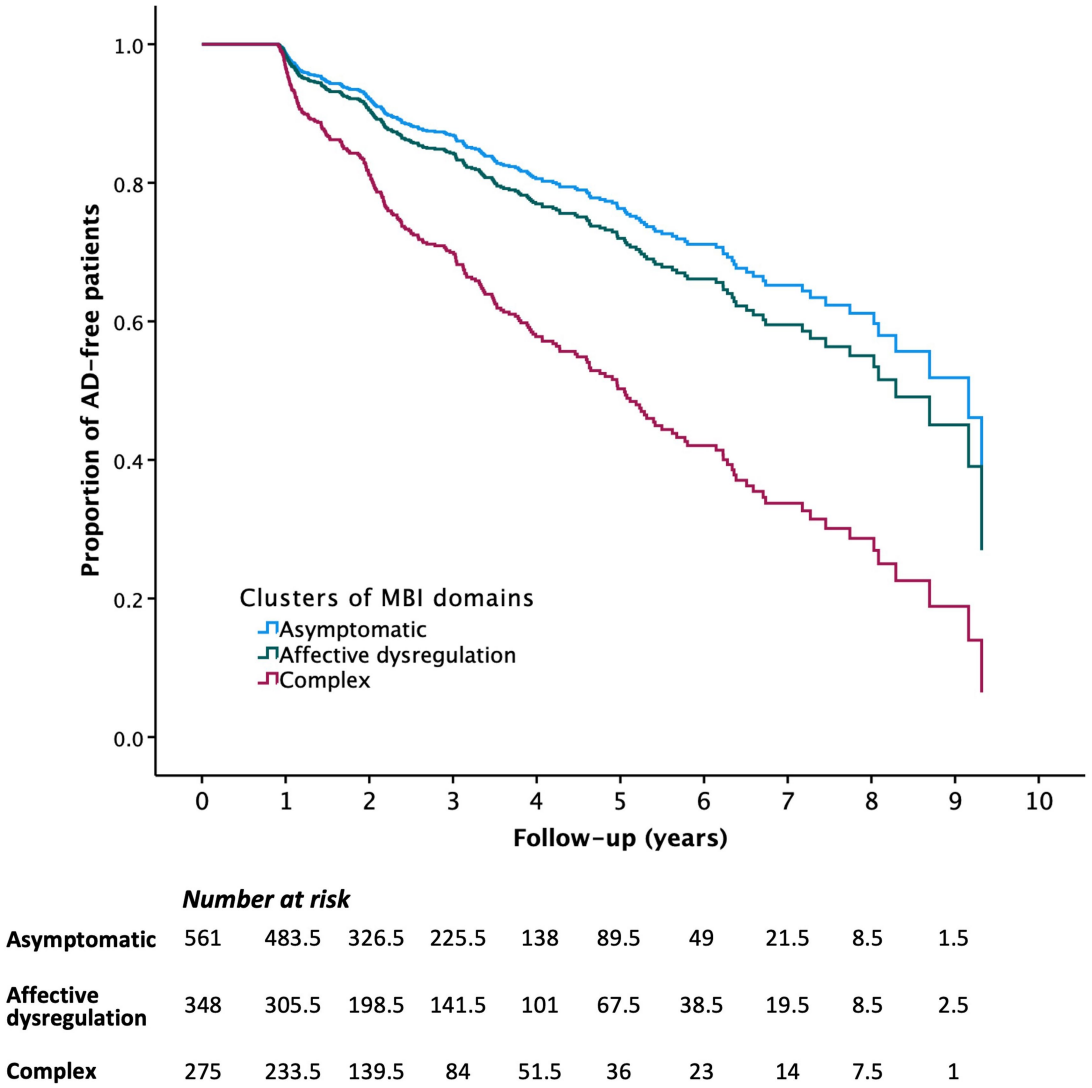
Survival curves of progression to Alzheimer’s disease by subgroups of study participants classified based on profiles of MBI domain symptoms.

Age (hazard ratio (HR) [95% confidence interval (CI)] = 1.097 (1.074–1.121), *p* < 0.001), years of education (HR [95% CI] = 0.963 (0.938–0.989), *p* = 0.005), MMSE (HR [95% CI] = 0.885 (0.857–0.914, *p* < 0.001) and SGDS scores (HR [95% CI] = 1.040 (1.007–1.075), *p* = 0.017) were significantly associated with the risk of progression to AD in univariate Cox regression analyses, and were used as covariates in the following Cox multivariate regression analyses. After adjusting the significant covariates, the presence of each MBI domain and MBI-positive were associated with higher risk of progression to AD (Table 4). Compared with the asymptomatic group (reference group), the complex group revealed a higher risk of progression to AD (HR [95% CI] = 2.541 [1.904–3.392], *p* < 0.001), but the affective dysregulation group did not (HR [95% CI] = 1.214 [0.883–1.670], *p* = 0.232) (Figure 1).

**Table 4:** Hazard ratios for progression to AD for MBI-positive and each MBI domain in multivariate Cox regression.

	Hazard ratio	95% CI	*p*-value
MBI-positive	1.783	1.365–2.328	< 0.001
Decreased motivation	2.131	1.595–2.848	< 0.001
Affective dysregulation	1.373	1.049–1.796	0.021
Impulse dyscontrol	1.920	1.444–2.554	< 0.001
Social inappropriateness	1.599	1.007–2.540	0.047
Abnormal perception/thought content	2.259	1.503–3.396	< 0.001

MBI, mild behavioral impairment; CI, confidence interval.

### Cortical thickness analysis

Demographic and clinical variables of the participants who scanned MRI are described in Table 5. There were no differences in age, sex, years of education, MMSE, follow-up time and rate of progression to AD among 3 groups. Two symptomatic groups had higher SGDS scores than the asymptomatic group.

**Table 5:** Demographic and clinical characteristics of the study participants with MRI scans classified based on profiles of MBI domain symptoms.

	Asymptomatic (1)	Affective dysregulation (2)	Complex (3)	*p*-vlaue^a^			
				3 groups	1 vs. 2	1 vs. 3	2 vs. 3
n	55	47	100				
Age, years	75.5 ± 4.8 (62–83)	74.4 ± 6.9 (61–85)	74.3 ± 5.5 (61–85)	0.382	0.555	0.373	0.993
Female:Male	34:21	29:18	58:42	0.862	0.990	0.643	0.670
Education, years	8.7 ± 5.3 (0–23)	8.2 ± 4.2 (0–16)	8.0 ± 5.4 (0–17)	0.680	0.865	0.655	0.964
MMSE	23.2 ± 3.8 (11–29)	23.0 ± 3.9 (10–30)	22.8 ± 3.7 (12–30)	0.793	0.964	0.782	0.936
SGDS	3.1 ± 2.9 (0–14)	7.1 ± 3.4 (1–13)	7.2 ± 4.5 (0–15)	< 0.001	< 0.001	< 0.001	0.989
Family history of dementia	11 (20.0)	8 (17.0)	23 (23.0)	0.697	0.700	0.666	0.407
Follow-up, years	2.5 ± 1.6 (0.9–7.6)	3.1 ± 2.4 (0.9–8.6)	2.7 ± 2.1 (0.9–9.2)	0.313	0.298	0.856	0.473
Progression to AD	18 (32.7)	23 (48.9)	42 (42.0)	0.244	0.096	0.257	0.430

Variables are presented mean ± standard deviation (range) or frequency (%). ^a^one-way analysis of variance with the Tukey *post-hoc* test for continuous variables and *χ*^2^ test for nominal variables. MMSE, mini-mental state examination; SGDS, short form of geriatric depression scale; MBI, mild behavioral impairment; AD, Alzheimer’s disease.

**Figure 2:**
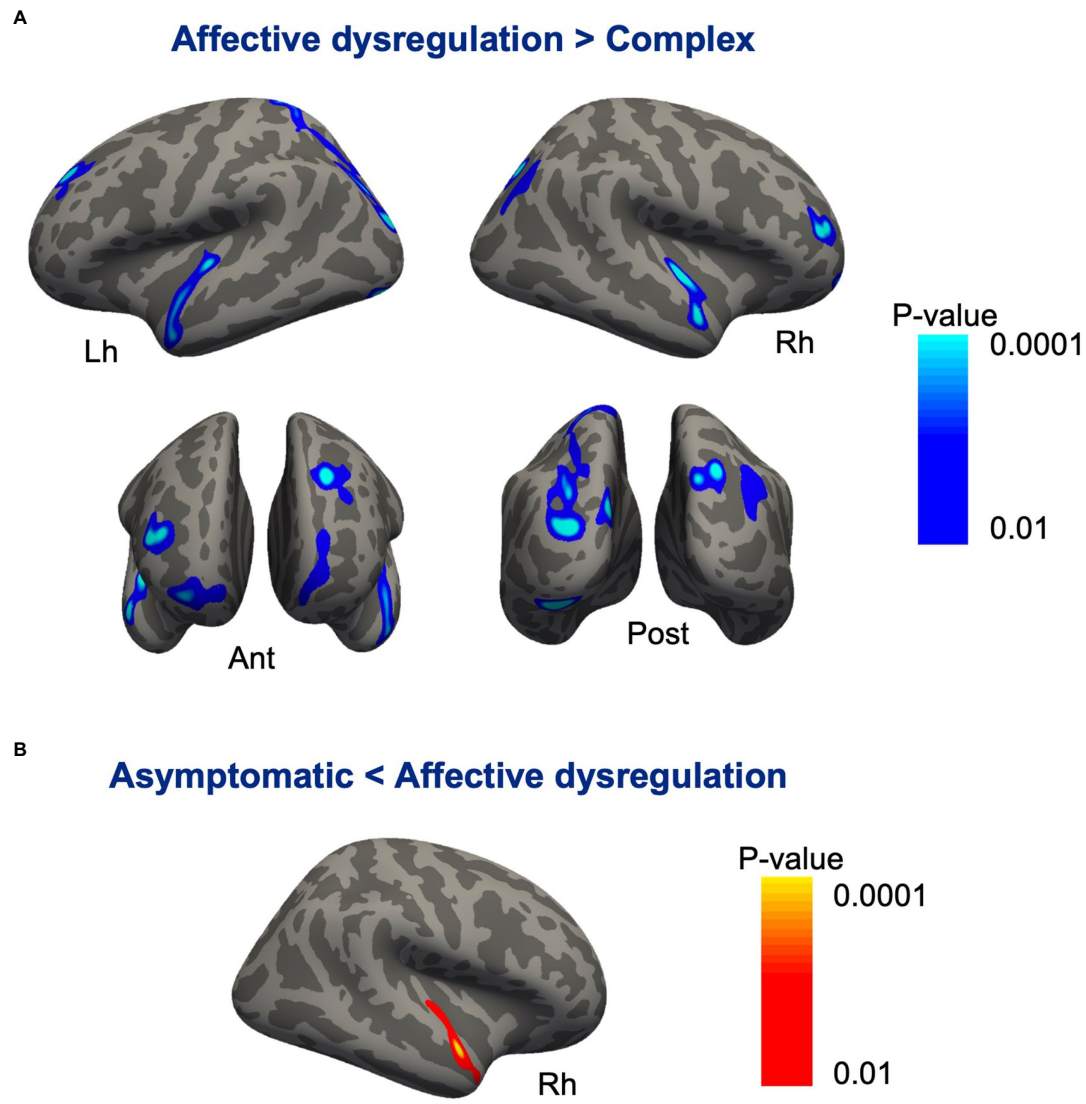
Cortical regions showing differences between the subgroups of pariticipants classified based on profiles of MBI domain symptoms. **(A)** Compared with the affective dysregulation group, the complex group revealed cortical thinning bilaterally in the inferior parietal cortex, lateral occipital cortex, lateral superior temporal gyrus, and frontopolar cortex. **(B)** The affective dysregulation group showed thicker cortex in the right superior temporal gyrus than the asymptomatic group.

Compared with the affective dysregulation group, the complex group revealed cortical thinning bilaterally in the inferior parietal cortex, lateral occipital cortex, lateral superior temporal gyrus, and the frontopolar cortex. We also found thinning in the left superior frontal sulcus, left cuneus and right middle frontal gyrus in the complex group (Figure 2A; Table 6). There were no significant differences in cortical thickness between the asymptomatic group and the complex group. While, the affective dysregulation group showed thicker cortex in right superior temporal gyrus than the asymptomatic group (Figure 2B; Table 6). In subcortical structures, there were no differences between 3 groups.

**Table 6:** Brain regions showing differences in cortical thickness between the subgroups of pariticipants classified based on profiles of MBI domain symptoms.

Region	Peak MNI coordinates (*x*, *y*, *z*)	Size (mm^2^)	Cluster-wise *p*-value
**Asymptomatic < Affective dysregulation**			
Right lateral superior temporal gyrus (BA 22)	56, 3, −12	603.69	0.0068
**Affective dysregulation > Complex**			
Left superior and transverse occipital sulci (BA 18)/ intraparietal sulcus (BA 39)/ superior parietal gyrus (BA 7)	−24, −89, 13	2208.14	0.0002
Left lateral superior temporal gyrus (BA 41/38)	−47, −19, 4	1031.05	0.0002
Left superior frontal sulcus (BA 8)	−19, 39, 35	544.14	0.0118
Left inferior occipital cortex (BA 18)	−30, −87, −16	470.54	0.0252
Left Cuneus (BA 19)	−5, −83, 31	454.92	0.0319
Left transverse frontopolar cortex (BA 10)	−20, 61, 10	441.91	0.0402
Right middle occipital gyrus/ angular gyrus (BA 39)	35, −69, 42	672.77	0.0028
Right circular sulcus of insular (inferior segment)/ lateral superior temporal gyrus (BA 22)	50, −15, −2	668.83	0.0034
Right superior occipital gyrus (BA 7)	23, −67, 34	650.76	0.0036
Right frontomarginal cortex (BA 10)	25, 57, −9	596.72	0.0076
Right middle frontal gyrus (BA 10)	39, 45, 13	585.81	0.0088
**Asymptomatic < Affective dysregulation**			
Right lateral superior temporal gyrus (BA 22)	56, 3, −12	603.69	0.0068

## Discussion

In the present study, we classified individuals with aMCI into 3 groups based on profiles of MBI domain symptoms and evaluated the differences in a risk of progression to AD and brain atrophy pattern between the groups. The complex group composed of aMCI patients with symptoms in multiple MBI domains showed significantly higher risk of progression to AD compared with the asymptomatic group. However, the risk of progression to AD in the affective dysregulation group composed of aMCI patients with affective dysregulation only did not differ with the asymptomatic group. Moreover, we found cortical thinning in the complex group in the bilateral temporal, parietal and frontal regions compared with the affective dysregulation group.

Prior studies reported significant associations between classes of NPS estimated using NPI-Q and progression to dementia as well as cognitive decline. In cognitively normal older adults, 4 classes of NPI were estimated: irritable, depressed, complex, and asymptomatic classes. All the three symptomatic classes were associated with greater hazard of progression to MCI or dementia compared with the asymptomatic class, with the complex class showing the greatest hazard. The complex class had high probabilities of almost all the NPI domains, particularly they had symptoms characteristic of both irritable class and depressed class (Leoutsakos et al., 2015). Similarly, in individuals with MCI, three latent clusters were identified based on patterns of NPI-Q: severe, affective, and asymptomatic. The affective and severe classes had a higher hazard of progression to dementia relative to the asymptomatic cluster. The severe class featured high rates of almost all symptoms, including agitation, anxiety, apathy, nighttime behaviors, and disinhibition (Forrester et al., 2016). When using three-factor model of NPI-Q: hyperactive, affective, and psychosis in individuals with MCI, affective and psychotic symptoms were associated with the risk of progression to dementia, but hyperactive symptoms were not. The risk was higher when affective and psychotic symptoms co-occurred (Liew, 2019). The previous studies consistently identified the symptomatic clusters of NPS were associated with progression to dementia, particularly complex or severe NPS clusters which featured multiple co-occurred NPS showed a higher risk of progression to dementia than other symptomatic clusters such as affective or irritability. These results are in line with our results that the complex group consisted of aMCI patients with symptoms in multiple MBI domains revealed the higher risk of progression to AD than the asymptomatic or affective dysregulation groups.

On the other hand, in the present study, we did not find the significant differences in the risk of progression to AD between the affective dysregulation group and the asymptomatic group. This seems unexpected findings, because the above mentioned previous studies using NPI-Q identified associations of the risk of progression to dementia with depressed and affective clusters, moreover, accumulating evidence have suggested that affective and emotional symptoms are risk factors of cognitive decline and dementia (Ismail et al., 2018). There are several possible explanations for the lack of associations between the affective dysregulation group and AD risk. First, it may be relatd with the NPS profiles in the affective dysregulation group. The affective dysregulation group was mostly composed of aMCI patients with depression (*n* = 134, 38.5%) or anxiety (*n* = 17, 4.9%) or both (*n* = 197, 56.6%). There were only 12 participants (1.0%) with elation/euphoria. By contrast, the depression or affective clusters of the previous studies included other NPS such as apathy (Leoutsakos et al., 2015; Liew, 2019) and irritability (Forrester et al., 2016) with high probabilities, in addition to depression and anxiety. Similary, previous studies identified relationships between depression or anxiety and dementia risk using NPI-Q items did not considered the co-occurrence of other items in NPI-Q (Somme et al., 2013; Geda et al., 2014; Gonzales et al., 2017). A more recent study using NPI-Q identified anxiety/depression class separately from irritability and apathy classes in individuals with MCI. When compared with asymptomatic class, irritability and apathy classes were associated with progression to dementia, but anxiety/depression class was not (Roberto et al., 2021). These findings suggest that anxiety/depression alone may not be sufficient to find association between NPS and progression to dementia and that considering co-occurrence of NPS is one of the important factor for understand relationships between NPS and progression to AD in MCI patients.

Second, the relationships between affective dysregulation and dementia risk could be affected by methodological differences, particularly by how the symptoms are defined. In the present study, MBI domains were approximated using the NPI-Q without considering the severity and frequency. Moreover, the NPI-Q does not clarify onset or chronicity of the symptoms. The presence of significant depressive symptoms defined by the GDS was a significant predictor of progression to AD in MCI patients, moreover, score increase on the scale was also associated with increased hazard of progression (Van der Mussele et al., 2014). A more recent large cohort study of cognitively unimpaired men found the graded association between the severity of depressive symptoms and the risk of dementia, with the risk being more pronounced for men with severe depression (Almeida et al., 2017). These results showed that severity of depressive symtpoms is one of the important factor determing the relationship between depression and dementia risk. In the present study, because we focused on the presence of MBI domains, the SGDS score was adjusted for multivariate Cox regression analyses. To confirm the effect of severity of depressive symptoms, we additionally dichotomized the affective dysregulation group using the SGDS score. When the presence of significant depressive symptoms was defined by the SGDS as a total score > 6, most of the affective dysregulation group (*n* =  285, 81.9%) showed the significant depressive symptoms. The affective dyregulation group with significant depressive symptoms did not reveal higher risk of progression to AD (HR = 1.237 [0.850–1.798], *p* = 0.266) compared with the asymptomatic group without significant depressive symptoms (*n =* 326, 58.1%). These findings suggest that the severity of depressive symptoms was not associated with the risk of AD in our study participants. However, since the current sample is limited to aMCI patients and the severity was evaluated using only SGDS, it should be careful about generalizing with our findings. It is also important to clarify whether the chronicity of symptoms are associated with progression to AD. Lee et al. identified that depressive symptoms evaluated using NPI-Q at baseline were not significantly associated with higher risk of AD development and increased cognitive decline in MCI subjects. However, those with depressive symptoms lasting more than 2 years had a higher conversion rate to AD and more cognitive decline (Lee et al., 2012). Moreover, MCI patients with chronic subsymdromal symptoms of depression over 4-year period were revealed accelerated cognitive decline (Gonzales et al., 2017). Although we evaluated NPI-Q at every visit during the follow-up period, it was difficult to consider the chronicity of symptoms due to the varying visit interval and follow-up time.

Lastly, previous studies have suggested that depression and anxiety symptoms are associated with subjective memory complaints rather than objective memory performances. In healthy population cohorts, subclinical depressive symptoms revealed a robust relationship with self-reports memory complaints, but not with performance on the standardized measure of memory (Schweizer et al., 2018). Individuals with MCI were significantly associated with anxiety and depression, but the odds of experiencing anxiety and depression were not increased in people with normal cognition or in those meeting criteria for MCI without subjective memory complaints (Yates et al., 2015, 2017). Moreover, symptoms of depression at follow-up were significantly associated with a change in cognitive status from cognitively normal to MCI over 2 years, but symptoms of depression and anxiety at baseline were not associated with cognitive status changes (Yates et al., 2017). These results suggest that we cannot ignore the possibility that anxiety and depression (affective dysregulation) could be experienced as a reaction to the awareness of cognitive decline rather than associated with neurobiological mechanisms underlying neurodegeneration in our aMCI patients.

When we evaluated whether the presence of individual MBI domain symptoms predicted progression to AD, all 5 domains were significant predictors, with abnormal perception/thought content having the greatest risk. However, the abnormal perception/thought content revealed the lowest prevalence with 4.4% among 5 domains. On the contrary, the affective dysregulation domain was the most prevalent MBI domain in the present study (47.3%), but the hazard ratio of progression to AD (1.37) was the lowest among 5 domains. One study with a large cohort of cognitively unimpaired controls found same tendency. The abnormal perception/thought content had the greatest effect on progression to AD, followed by the other 4 domains, but the prevalence of this domain was the lowest among 5 domains (Ruthirakuhan et al., 2022). In a study with MCI, only abnormal perception/thought content domain among 5 domains was a significant risk factor for progression to AD, but this domain symptom was present in only 3.0% of participants (Yokoi et al., 2019). On the other hand, when we analyzed the subgroups of aMCI patients classified based on profiles of MBI domain symptoms, the complex group showed more than 2.5 times the hazard of progression to AD compared with the asymptomatic group, with the prevalence of 23.2%. Collectively, our findings suggest that identifying the subgroups of participants using MBI domain profiles might be more sensitive prognostic marker of AD than individual MBI domains which could miss significant cases in the preclinical phase of disease.

In the cortical thickness analyses, the complex group revealed cortical thinning bilaterally in the inferior parietal cortex, lateral occipital cortex, lateral superior temporal cortex, and frontopolar cortex compared with the affective dysregulation group. Previous studies have consistently reported associations between the frontal, temporal, parietal lobes and NPS in patients with MCI as well as those with AD. NPS including delusion, hallucination, wandering, and agitation were associated with accelerated atrophy in the lateral frontal and lateral parietal lobes in patients with MCI or mild AD (Rafii et al., 2014). When NPS were divided into hyperactivity, psychosis, affective and apathy subsyndromes using NPI, 3 subsyndromes excluding affective subsyndrome were associated with atrophy in the lateral prefrontal and lateral temporal cortex in MCI and AD subjects (Misquitta et al., 2020). Greater agitation and aggression in MCI-converter and AD patients were associated with greater atrophy in the frontal and limbic areas (Trzepacz et al., 2013). Also, aMCI patients with chronic depressive symptoms including depression/dysphoria, apathy/indifference, and appetite/eating disturbances showed accelerated cortical atrophy in the frontal lobe and anterior cingulate cortex (Sacuiu et al., 2016). In this regard, the cortical thinning in the complex group in the present study may be due to their multiple and severe NPS compared with the affective dysregulation group. Moreover, the lateral temporal cortex and inferior parietal cortex are included in the AD cortical signature that is associated with AD symptoms severity, tau pathology and a risk of progression to AD (Bakkour et al., 2009; Dickerson et al., 2009; McEvoy et al., 2009; Wang et al., 2015), which is in line with the our finding of a higher risk of progression to AD in the complex group. Therefore, the cortical thinning in the complex group may also support the possibility that aMCI patients with complex NPS are in a more advanced stage of AD continuum and are more likely to progress to dementia than those with only affective dysregulation.

Of note, the affective dysregulation group revealed relatively thicker right superior temporal cortex compared with the asymptomatic group. Although it was not statistically significant, the brain cortical regions showing atrophy in the complex group were relatively thicker in the affective dysregulation group than the asymptomatic group. The relative cortical thickening in the affective dysregulation group may reflect a compensatory effect associated with additional recruitment of brain regions for attenuate their mood symptoms or inflammation. Patients with dysthymic disorder had diffusely thicker cortices than healthy controls, especially in the lateral temporal, inferior parietal, and cingulate cortex. The cortical thickening correlated inversely with depressive symptom severity, and declined toward values to healthy controls during the trial of antidepressant medication, suggesting a compensatory hypertrophy of the cortex (Bansal et al., 2018). Patients with first-episode medication-naïve major depressive disorder revealed cortical thickening in the inferior parietal and medial orbitofrontal cortices (Qiu et al., 2014; Yang et al., 2015), but the pattern of thickening disappeared or inversed as the disease progressed (Suh et al., 2019). Previous studies identified that major depressive disorder is accompanied by immune dysregulation, which suggest that the cortical thickening in the early course of the disorder could be associated with increased immune activation in brain such as cellular hypertrophy and cytokine-activated astrocyte proliferation (Li et al., 2020).

The cortical thickening was also appeared in the earliest stages of AD pathology. When classifying cognitively unimpaired participants into preclinical AD stages: Aß-negative/p-tau-negative, Aß-positive/p-tau-negative, and Aß-positive/p-tau-positive, individuals with the Aß+/p-tau– revealed cortical thickening in the middle temporal, superior and inferior temporal, and lateral occipital regions compared with the Aß−/p-tau– group, but the Aß+/p-tau+ group showed relatively thinner cortex in these brain regions than the Aß−/p-tau– group (Fortea et al., 2014; Montal et al., 2018). It has been suggested that the cortical thickening in the early asymptomatic stage of AD may associated with amyloid-induced neuroinflammation. Indeed, higher levels of progranulin (PGRN) in the CSF, markers of microglial activation, correlated with thicker cortex in the bilateral frontoparietal, occipital and right temporal areas in cognitively unimpaired subjects with Aß+/p-tau–. However, the correlations were not found in the cognitively normal individuals with Aß−/p-tau– or Aß+/p-tau+ (Batzu et al., 2020). Also, another CSF biomarker of microglial activation, soluble fragment of the triggering receptor expressed on myeloid cells (sTREM2) revealed positive correlations with gray matter volumes in the bilateral inferior and middle temporal, precuneus, and inferior parietal regions in MCI due to AD, but AD or control subjects did not show the correlations (Gispert et al., 2016). Although it would be speculative because we did not evaluate neuropathological and neuroinflammation biomarkers in the present study, the relative thickening of the affective dysregulation group may reflect early amyloid-induced neuroinflammatory mechanisms in development of disease without positive on tau. The complex group, on the other hand, may be associated with more advanced stages of disease in which the pattern of thickening was disappeared or inversed. In line with these hypotheses, a recent study found that higher MBI-C scores were associated with higher tau-PET signal and CSF p-tau levels in Aß-positive cognitively unimpaired subjects (Johansson et al., 2021). Further studies of relationships between MBI and dementia risk in the context of A/T/N classification scheme (Jack et al., 2016) could confirm whether the MBI is proper tool for AD risk stratification in MCI and whether the structural changes we observed are based on AD pathology.

There are several limitations in the current study. We used the NPI-Q to approximate MBI. The NPI-Q utilizes reference time of 1-month, whereas MBI criteria requires a 6-month reference range. Therefore, there are possibility of overestimation of the prevalence of MBI. We found 52.6% of aMCI patients were MBI-positive, which was similar to or lower than previous studies using NPI-Q to approximate MBI in MCI patients (Sheikh et al., 2018; Yokoi et al., 2019). However, the MBI prevalence in MCI patients was 14.2% when the diagnosis of MBI was made *via* a semi-structured independent interview in accordance with ISTAART-AA criteria (Mallo et al., 2018). Our study should be replicated with stricter way to diagnosis of MBI, such as the MBI-C (Ismail et al., 2017). The MBI-C is a rating scale designed to elicit emergent NPS in the older population, in accordance with the MBI criteria. A recent study found that the correlations between NPI-Q and MBI-C were moderate to high for total and domain scores, and both scales were associated with scores of the Montreal cognitive assessment. However, the MBI-C generated more conservative estimates for capturing prevalence of MBI (Hu et al., 2022). Another important limitation is the lack of Aß and tau biomarkers, which limits interpretion of relationships between structural changes and AD pathology in the present study. Other important risk factors of progression to AD, such as APOE genotypes and cardiovascular disease, were not considered for survival analyses. Moreover, although we adopted the standardized clinical diagnostic criteria and diagnosis was made at consensus conferences attended by psychiatrists and neuropsychologists, the diagnosis of AD was not confirmed neuropathologically. Finally, the sample size of brain structural analyses was relatively small.

In summary, the multiple co-occurring NPS that constitute MBI domains are associated with a higher risk of progression to AD and cortical thinning in the frontal, temporal, and parietal regions in individuals with aMCI. However, affective dysregulation only was not the predictor of progression to AD. These results support the possibility that the MBI, particularly the multiple co-occurring MBI domains, is the important risk marker of progression to AD in aMCI patients. Therefore, evaluation of MBI could be useful for risk stratification for AD and appropriate intervention in MCI individuals.

## Data availability statement

The original contributions presented in the study are included in the article/supplementary material, further inquiries can be directed to the corresponding author.

## Ethics statement

The studies involving human participants were reviewed and approved by Institutional Review Board of SMG-SNU Boramae Medical Center. The patients/participants provided their written informed consent to participate in this study.

## Author contributions

EY designed the study and executed the data analysis, and wrote the first draft of the manuscript. J-YL and YK reviewed the data analysis and revised the manuscript critically for important intellectual content. J-YL and SK conducted major role in the acquisition data. All authors contributed with different roles in the present work, agreed with the presented findings, and approved the submitted version.

## Funding

This work was supported by the National Research Foundation of Korea (NRF) grant funded by the Korea government (MSIT) (no. 2021R1C1C2005543) and by the Ministry of Education, Science and Technology (MEST) (NRF-2018R1A5A2025964).

## Conflict of interest

The authors declare that the research was conducted in the absence of any commercial or financial relationships that could be construed as a potential conflict of interest.

## Publisher’s note

All claims expressed in this article are solely those of the authors and do not necessarily represent those of their affiliated organizations, or those of the publisher, the editors and the reviewers. Any product that may be evaluated in this article, or claim that may be made by its manufacturer, is not guaranteed or endorsed by the publisher.
